# Targeting long non-coding RNA-TUG1 inhibits tumor growth and angiogenesis in hepatoblastoma

**DOI:** 10.1038/cddis.2016.143

**Published:** 2016-06-30

**Authors:** R Dong, G-B Liu, B-H Liu, G Chen, K Li, S Zheng, K-R Dong

**Affiliations:** 1 Department of Pediatric Surgery, Children's Hospital of Fudan University, Shanghai, China; 2 Shanghai Key Laboratory of Birth Defect, Shanghai, China; 3 Key Laboratory of Neonatal Disease, Ministry of Health, Shanghai, China

## Abstract

Hepatoblastoma is the most common liver tumor of early childhood, which is usually characterized by unusual hypervascularity. Recently, long non-coding RNAs (lncRNA) have emerged as gene regulators and prognostic markers in several cancers, including hepatoblastoma. We previously reveal that lnRNA-TUG1 is upregulated in hepatoblastoma specimens by microarray analysis. In this study, we aim to elucidate the biological and clinical significance of TUG1 upregulation in hepatoblastoma. We show that TUG1 is significantly upregulated in human hepatoblastoma specimens and metastatic hepatoblastoma cell lines. TUG1 knockdown inhibits tumor growth and angiogenesis *in vivo*, and decreases hepatoblastoma cell viability, proliferation, migration, and invasion *in vitro*. TUG1, miR-34a-5p, and VEGFA constitutes to a regulatory network, and participates in regulating hepatoblastoma cell function, tumor progression, and tumor angiogenesis. Overall, our findings indicate that TUG1 upregulation contributes to unusual hypervascularity of hepatoblastoma. TUG1 is a promising therapeutic target for aggressive, recurrent, or metastatic hepatoblastoma.

Angiogenesis is essential for tumor development and metastasis. It participates in nutrient supply and waste exchange, and accelerates tumor growth. Angiogenesis also assists tumor cells to leave the primary site and enter circulation, which contributes to tumor metastasis.^1, 2^ Thus, preventing tumor angiogenesis is a promising tactic in inhibiting tumor progression and metastasis. Tumor angiogenesis is a very complicated process. Numerous signaling molecules and pathways have been identified to affect angiogenesis, such as vascular endothelial growth factor (VEGF), platelet-derived growth factor, basic fibroblast growth factor, non-coding RNAs, and their related pathways.^1^^,3, 4^ Understanding how these components functionally interact contributes to the identification of novel druggable targets.

Hepatoblastoma is the primary liver tumor in infants and toddlers, accounting for ~1% of pediatric cancers. It usually originates from immature liver precursor cells, and is histologically divided into epithelial or mixed epithelial/mesenchymal tissues.^5, 6, 7^ Surgical resection, adjuvant chemotherapy, and liver transplantation is the available method for treating hepatoblastoma. Further understanding the mechanism underlying hepatoblastoma progression is still required for improving diagnosis, prevention, and treatment.^5^ Hepatoblastoma is usually characterized by unusual hypervascularity.^8^ However, the mechanism orchestrating vascularization is still unclear in hepatoblastoma.

Long non-coding RNAs (lncRNAs) are non-coding transcripts >200 nucleotides in length. They play important roles in chromatin remodeling, transcription regulation, epigenetic regulation, and post-transcriptional mRNA processing.^9^ They are emerging as novel players in the cancer paradigm, demonstrating the potential roles in both oncogenic and tumor suppressive pathway.^10^ In our previous study, we performed genome-wide lncRNA analysis to identify hepatoblastoma-related lncRNAs. LncRNA-TUG1 is found to be differentially expressed between hepatoblastoma and normal liver tissue.^11^ In this study, we elucidate the biological and clinical significance of TUG1 upregulation in hepatoblastoma. We show that TUG1 knockdown significantly suppresses angiogenesis *in vitro* and *in vivo* and xenograft tumor growth in nude mice. TUG1/miR-34a-5p/VEGFA network is involved in regulating hypervascularity and hepatoblastoma progression.

## Results

### LncRNA-TUG1 is significantly upregulated in human hepatoblastoma specimens and cell lines

**Figure 1:**
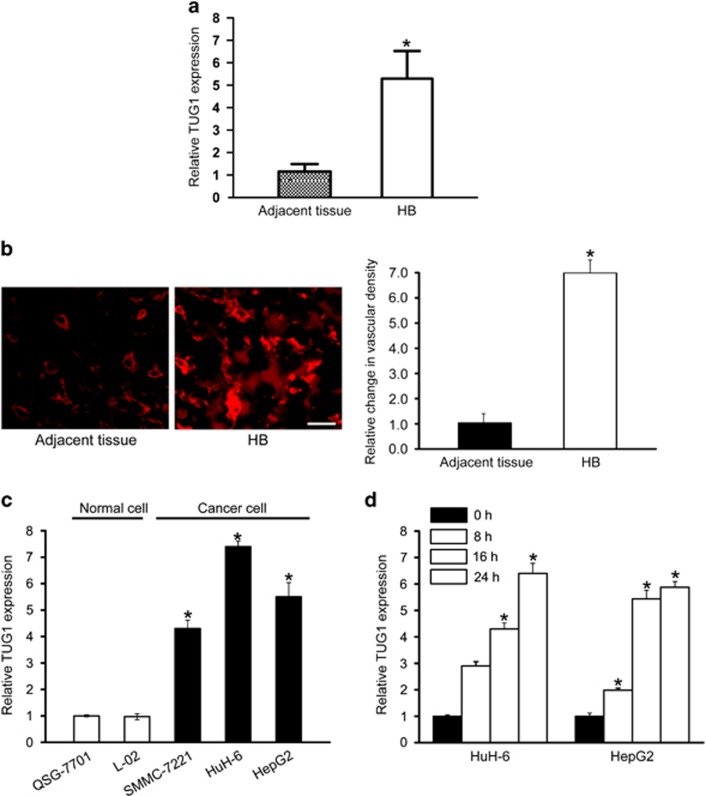
LncRNA-TUG1 is significantly upregulated in human hepatoblastoma specimens and cell lines. (**a**) Total RNAs were extracted from 10 human hepatoblastoma specimens (HB) and 10 matched adjacent non-cancerous liver tissues. qRT-PCRs were conducted to detect TUG1 expression. Data were from three independent experiments (Mann–Whitney *U*-test; *P*=0.0012; *P<0.05 versus adjacent tissue). (**b**) CD31 immunofluoresence staining was conducted to detect microvascular density in hepatoblastoma tissues (*n*=6) compared with the matched normal tissues (*n*=6). A representative image was shown. Scale bar, 100 *μ*m. Data were analyzed by Mann–Whitney *U*-test (*P*=0.0008; *P<0.05 versus adjacent tissue). (**c**) qRT-PCRs were conducted to detect TUG1 expression in nonmalignant QSG-7701 and L01 hepatocytes, malignant SMMC-7221, HuH-6, and HepG2 (*n*=4). Statistical differences were analyzed by Student's *t*-test (two-sided). *P*=0.0017 (SMMC-7221)*; P*=0.0009 (HuH-6); *P*=0.0016 (HepG2). *indicated significant difference compared with TUG1 expression in QSG-7701 cell. (**d**) Hepatoblastoma cell lines, HuH-6 and HepG2 cell, were exposed to hypoxic condition for the indicated time periods, respectively. qRT-PCRs were conducted to detect TUG1 expression (*n*=4). Statistical differences were analyzed by Student's *t*-test (two-sided). *P*=0.0032 (HuH-6, 16 h)*; P*=0.0016 (HuH-6, 24 h); *P*=0.0039 (HepG2, 8 h); *P*=0.0021 (HepG2, 16 h); *P*=0.0019 (HepG2, 24 h). *indicated significant difference compared with TUG1 expression at 0 h

We previously show that lncRNA-TUG1 is abnormally expressed in hepatoblastoma specimens.^11^ Here, we further collected human specimens to detect TUG1 expression pattern in hepatoblastoma. TUG1 was significantly upregulated in hepatoblastoma tissues compared with the matched normal tissues (Figure 1a). Meanwhile, we detected tumor angiogenesis by CD31 immunofluoresence staining of tumor sections. Microvascular density was significantly higher in hepatoblastoma tissues compared with the matched normal tissues (Figure 1b). TUG1 upregulation was also observed in all examined hepatocellular carcinoma and hepatoblastoma cells compared with the nonmalignant QSG-7701 and L01 hepatocytes (Figure 1c). Hypoxia is a pathological factor involved in tumorigenesis. We exposed hepatoblastoma cells to hypoxic condition to mimic tumor microenvironment. TUG1 expression was found to be progressively upregulated (Figure 1d). Collectively, these results suggest that TUG1 is a potential regulator involved in the tumorigenesis of hepatoblastoma.

### LncRNA-TUG1 knockdown inhibits tumor growth and tumor angiogenesis *in vivo*

**Figure 2:**
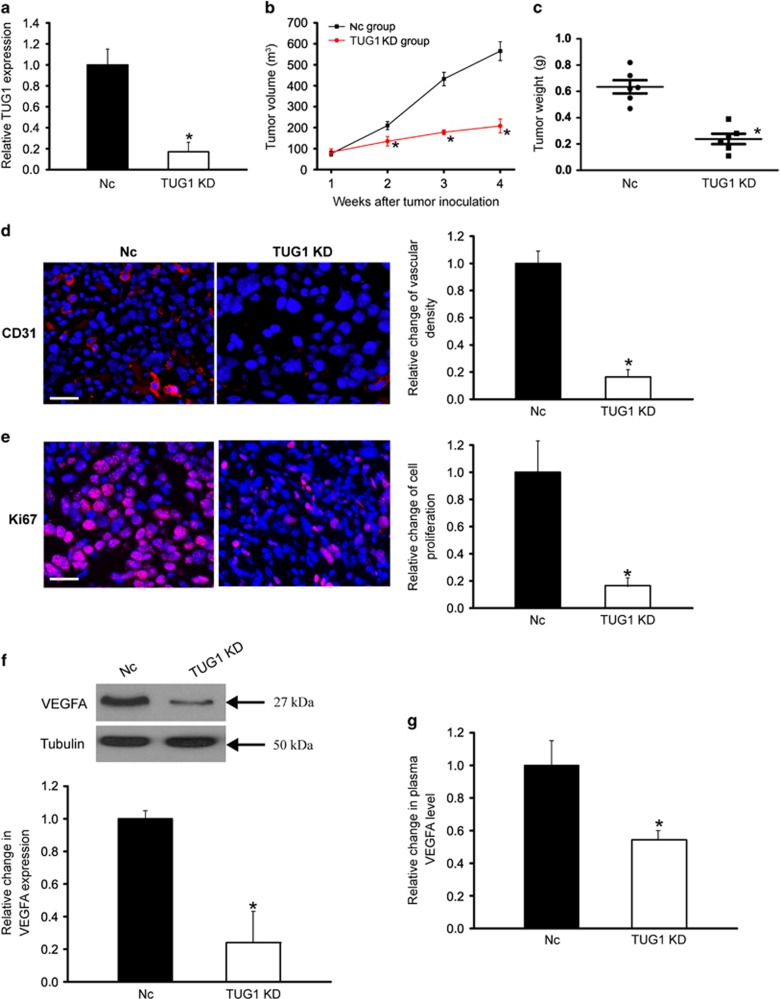
LncRNA-TUG1 knockdown inhibits tumor growth and angiogenesis *in vivo*. An *in vivo* hepatoblastoma model was established by injection of stable TGU1 knockdown HuH-6 cells and scrambled shRNA-transfected HuH-6 cells. (**a**) TUG1 levels in tumors isolated from the TUG1 knockdown (KD) group and negative control group (Nc) were determined by qRT-PCRs (Student's *t*-test, *P*=0.0011). (**b**) Comparison of tumor growth between TUG1 KD group and Nc group. The volume of solid tumor was measured every week using a vernier caliper. Tumor volume (V) was calculated using the formula, *V*= (*L* × *W*^2^) × 0.5 (*n*=6; Mann–Whitney *U*-test; *P*=0.0362 (2w), *P*=0.0252 (3w)*, P*=0.0182 (4w)). (**c**) The tumors were weighed immediately after isolation from mice. Tumor weight was plotted between the two groups (Mann–Whitney *U*-test; *P*=0.0089). (**d** and **e**) CD31 and Ki67 immunofluoresence staining was conducted to detect microvascular density and cell proliferation in hepatoblastoma tissues from TUG1 KD group and Nc group. A representative image and statistical result was shown (*n*=6; Mann–Whitney *U*-test; *P*=0.0045 (**d**) and *P*=0.0034 (**e**)). Scale bar: 100 *μ*m. (**f**) Western blots were used to compare VEGFA expression between TUG1 KD group and Nc group. Tubulin expression was detected as the loading control. VEGFA expression was determined as the ratio of densitometric value compared with Tubulin expression. A representative immunoblot was shown along with the quantitative data (*n*=6; Mann–Whitney *U*-test; *P*=0.0112). (**g**) ELISAs were conducted to compare the difference in plasma VEGFA levels between TUG1 KD group and Nc group (*n*=6; Mann–Whitney *U*-test; *P*=0.0165). *indicated significant difference between Nc group and TUG1 KD group

To reveal the role of TUG1 expression change in hepatoblastoma *in vivo*, we used a mouse hepatoblastoma xenograft model to determine the effect of TUG1 knockdown on tumor growth and angiogenesis. Stable TGU1 knockdown HuH-6 cells or scrambled shRNA-transfected HuH-6 cells were injected subcutaneously into the right flank of immunocompetent mice (C57BL/6). We found that TUG1 levels were significantly downregulated in stable TUG1 knockdown group (Figure 2a). Tumor size was significantly reduced in the TUG1 knockdown group 2 weeks after hepatoblastoma inoculation. At the end of the experiment, tumor volume was reduced by 28% in TUG1 knockdown group compared with the control group (Figure 2b), which was consistent with tumor weight reduction (Figure 2c).

Tumor growth is tightly dependent on tumor angiogenesis, which can provide an exchange of nutrients, oxygen, and paracrine stimuli to the tumor.^12^ CD31 immunofluorescence staining showed that subcutaneous xenograft tumors in TUG1 knockdown group had lower microvessel density (Figure 2d). Ki67 staining showed that TUG1 knockdown significantly decreased the proportion of proliferating (Ki67^+^) tumor cells (Figure 2e). VEGF is an important regulator of tumor angiogenesis. Western blots and enzyme-linked immunosorbent assay (ELISA) experiments showed that TUG1 knockdown significantly decreased VEGFA levels in hepatoblastoma tissue and blood (Figures 2f and g).

### LncRNA-TUG1 knockdown leads to decreased cell viability, proliferation, invasion, and migration *in vitro*

**Figure 3:**
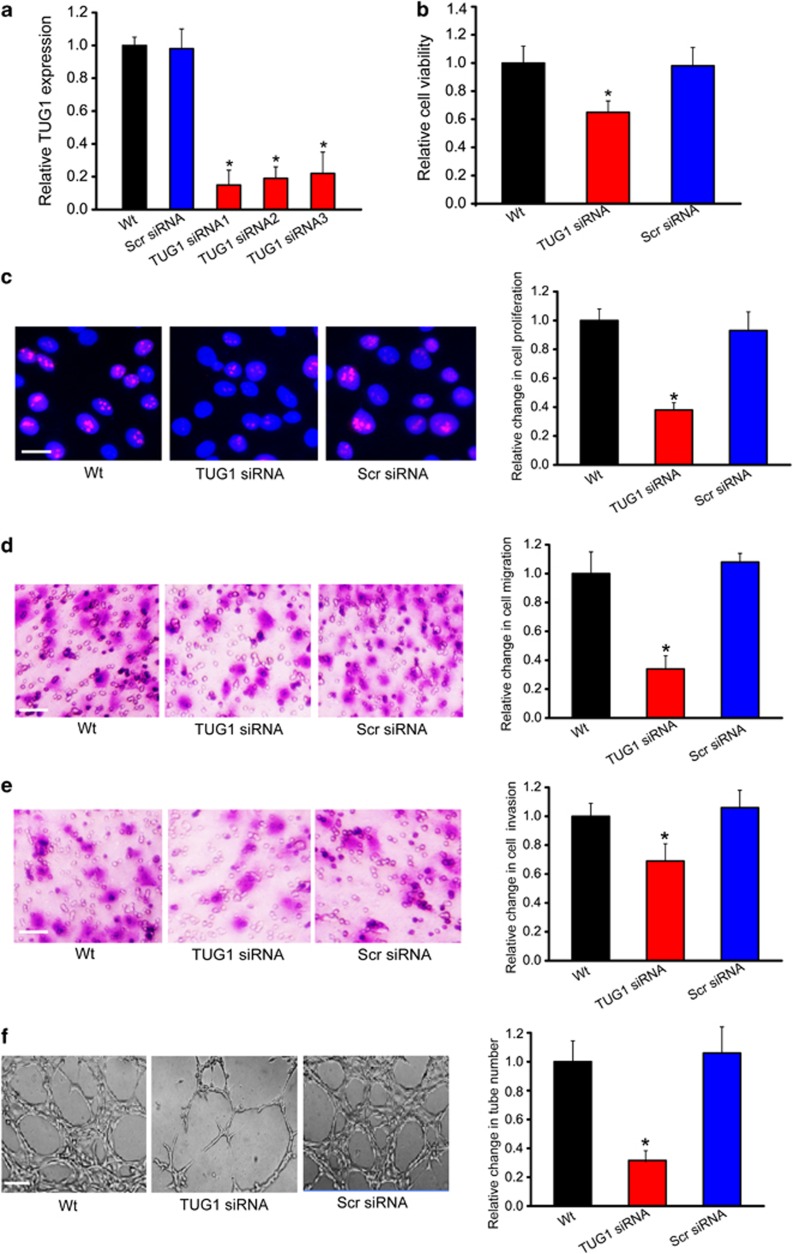
LncRNA-TUG1 knockdown results in decreased cell viability, proliferation, invasion, and migration *in vitro.* (**a**) HuH-6 cells were transfected with scrambled siRNA (Scr), TUG1 siRNA1, TUG1 siRNA2, TUG1 siRNA3, or left untreated (WT) for 48 h. qRT-PCRs were conducted to detect TUG1 levels. The data were shown as fold increase compared with WT group. '*' indicated significant difference compared with WT group (*n*=4; Student's *t*-test; *P*=0.0105, 0.0198, and 0.0228). (**b–e**) HuH-6 cells were transfected with scrambled (Scr) siRNA, TUG1 siRNA, or left untreated for 48 h. Cell viability was detected using MTT method (**b**; *n*=4; Student's *t*-test; *P*=0.0375). Ki67 immunofluorescence staining and quantitative analysis showed that TUG1 knockdown reduced HuH-6 cell proliferation. Scale bar, 10 *μ*m (**c**; *n*=4; Student's *t*-test; *P*=0.0165). Transwell assays showed that TUG1 knockdown decreased HuH-6 cell migration and invasion ((**d**; *n*=4; Student's *t*-test; *P*=0.0119) and (**e**; *n*=4; Student's *t*-test; *P*=0.0243)). (**f**) HUVECs were cultured in 24-well plates coated with matrigel in TCM derived from HuH-6 cells transfected with TUG1 siRNA, or a negative control siRNA, or without any transfection, and added to matrigel-coated wells. Representative images of tube formation and the number of tube formation were shown (*n*=4; Student's *t*-test; *P*=0.0139). Scale bar: 50 *μ*m

To reveal the functional significance of TUG1 alteration in hepatoblastoma *in vitro*, we first designed three different TUG1 siRNAs. TUG1 siRNA transfection significantly reduced TUG1 expression in HuH-6 cells (Figure 3a). We selected TUG1 siRNA with the greatest silencing efficiency for the subsequent study. MTT assay showed that TUG1 knockdown decreased HuH-6 cell viability (Figure 3b). Ki67 immunofluorescence staining showed that TUG1 knockdown reduced the proliferation of HuH-6 cells (Figure 3c). We then conducted transwell assay to investigate the effect of TUG1 knockdown on the migration and invasion of HuH-6 cells. TUG1 knockdown significantly decreased the migration and invasion of HuH-6 cells compared with the scrambled siRNA-transfected group (Figures 3d and e). We also used HepG2 hepatoblastoma cell line to investigate the functional significance of TUG1 alteration *in vitro*. TUG1 knockdown significantly reduced the viability, proliferation, migration, and invasion of HepG2 cells (Supplementary Fig. S1).

To further explore the biological significance of TUG1 in tumor angiogenesis, we carried out matrigel-based capillary tube-formation assays by human umbilical vein endothelial cells (HUVECs) using the tumor cell-conditioned medium (TCM) from HuH-6 cells. TCM was collected from wells with HuH-6 cells transfected with TUG1 siRNA, or a negative control siRNA, or without any transfection, and added to matrigel-coated wells. HUVECs were then plated into the wells to form capillary tubes. Less capillary tube formation was found for HUVECs in the presence of TCM derived from cells transfected with TUG1 siRNA (Figure 3f).

### LncRNA-TUG1 functions as miR-34a-5p sponge in hepatoblastoma cell

**Figure 4:**
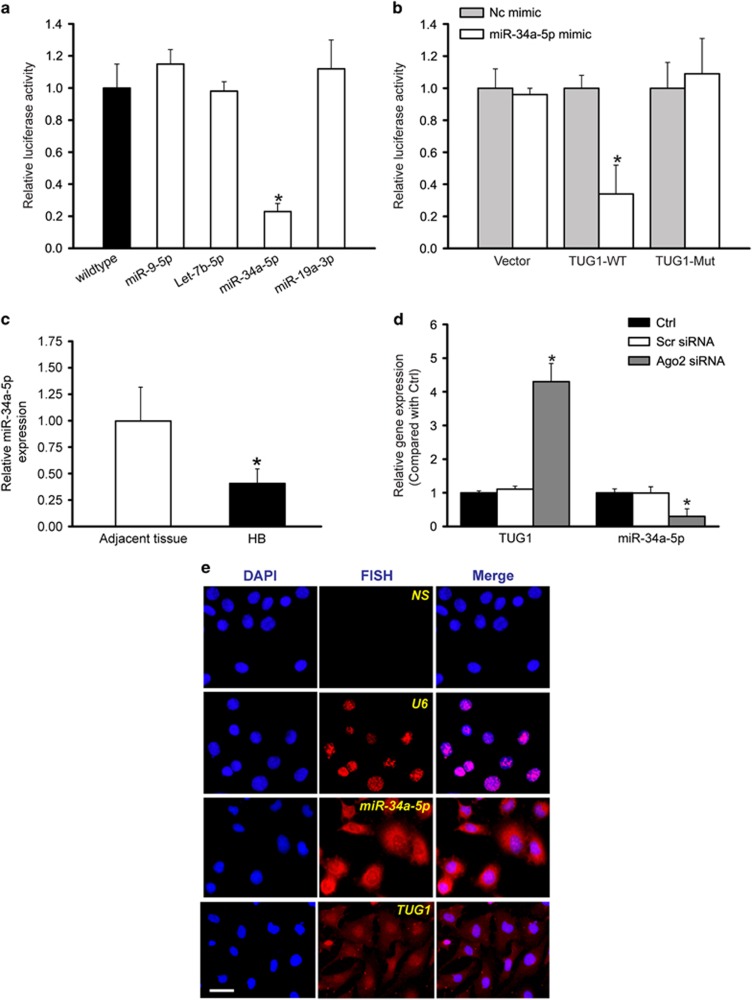
LncRNA-TUG1 functions as miR-34a-5p sponge in hepatoblastoma cell. (**a**) HuH-6 cells were co-transfected RLuc-TUG1-WT with different miRNA mimics. Luciferase activity was detected using the dual luciferase assay (Promega). The group was only transfected with RLuc-TUG1-WT vector as the control group. Luciferase activity was detected 48 h after transfection (*n*=4; Student's *t*-test; *P*=0.0189; *P<0.05 versus wildtype group). (**b**) RLuc-TUG1-WT or RLuc-TUG1-Mut was co-transfected with miR-34a-5p mimic into HuH-6 cells in parallel with the vector. Luciferase activity was detected 48 h after transfection. The data were shown as the relative change compared with the control group (*n*=4; Student's *t*-test; *P*=0.0222; *P<0.05 Nc mimic group versus miR-34a-5p mimic group). (**c**) Total RNAs were extracted from 10 human hepatoblastoma specimens and 10 matched adjacent non-cancerous liver tissues. qRT-PCRs were conducted to detect the relative expression of miR-34a-5p (*n*=4; Mann-Whitney *U*-test; *P*=0.0138; *P<0.05 versus adjacent tissue). (**d**) HuH-6 cells were transfected with Ago2 siRNA, scrambled siRNA, or left untreated (Ctrl). miR-34a-5p or TUG1 levels were detected using qRT-PCRs (*n*=4; Student's *t*-test; *P*=0.0104 and *P*=0.0261). *indicated significant difference compared with Ctrl group. (**e**) RNA-fluorescent *in situ* hybridization (FISH) for miR-34a-5p and TUG1 in HuH-6 cells. Nuclei, blue; miR-34a-5p, red. U6 was detected as the positive control, whereas NS indicated no probe addition. Scale bar, 10 *μ*m

LncRNAs could act as miRNA sponges, and regulate the availability of miRNA for binding mRNA targets.^13^ To determine whether lncRNA-TUG1 functions as a miRNA sponge, we first used the DIANA-LncBase database to search for potential miRNA recognition elements on lncRNA-TUG1.^14^ miR-9-5p, miR-34a-5p, Let-7b-5p, and miR-19a-3p was predicated as the potential miRNA targets on TUG1. Subsequently, TUG1 cDNA was cloned into the downstream of luciferase gene (RLuc-TUG1-WT) and transfected into HuH-6 cells with different miRNA mimics. We found that the activity of RLuc-TUG1-WT was significantly reduced by miR-34a-5p mimic, but not by other miRNA mimics (Figure 4a). The fragment 5′-UAGCUGG-3′ of miR-34a-5p pairs well with the fragment 5′-CCAGCUA-3′ located at the 1584-1612 nt of lncRNA-TUG1. To avoid unspecific binding, we also mutated the miR-34a-5p binding site of TUG1, from 'CCAGCUA' to 'AACAAGC-3', to generate RLuc-TUG1-Mut. miR-34a-5p mimic transfection significantly reduced RLuc-TUG1-WT activity, but had no effect on RLuc-TUG1-Mut activity (Figure 4b). These data suggest that miR-34a-5p directly regulates TUG1 expression in HuH-6 cells. We also compared miR-34a-5p expression pattern between hepatoblastoma and the matched normal tissues. miR-34a-5p was found to be downregulated in hepatoblastoma tissues (Figure 4c), showing an opposite expression trend compared with TUG1 expression pattern.

Ago2 is a key component of RNA-induced silencing complex that binds miRNA complexes to mRNA targets.^15^ We also studied whether TUG1 expression is regulated by miRNAs via Ago2 knockdown *in vitro*. Ago2 knockdown resulted in a significant increase in TUG1 expression, whereas miR-34a-5p stability was impaired by Ago2 knockdown (Figure 4c). We further conducted *in situ* hybridization to investigate miR-34a-5p expression. miR-34a-5p was expressed both in the cytoplasm and nucleus of HuH-6 cells (Figure 4d), implying that miR-34a-5p has the potential for TUG1 regulation both in the cytoplasm and nucleus.

### LncRNA-TUG1 regulates the expression of miR-34a-5p target gene, VEGFA, in hepatoblastoma cell

**Figure 5:**
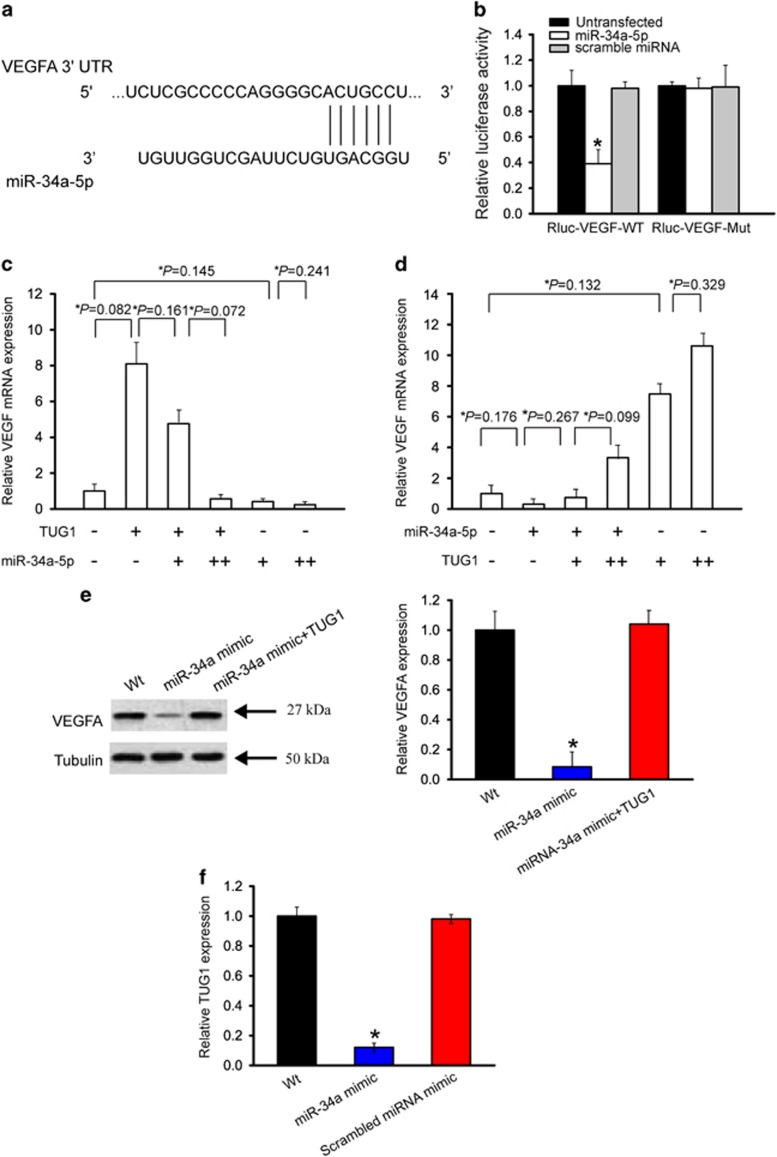
LncRNA-TUG1 regulates the expression of miR-34a-5p target gene, VEGFA, in hepatoblastoma cell. (**a**) Computational miRNA target prediction analysis revealed that the fragment 5′-GGCAGU-3′ of miR-34a-5p pairs well with the fragment 5′-ACUGCC-3′ located at VEGFA 3'-UTR. (**b**) VEGFA (RLuc-VEGFA-WT) and mutant (RLuc-VEGFA-Mut) were co-transfected with miR-34a-5p, scrambled miRNA, or left untreated. Luciferase activity was detected using the dual luciferase assay (*n*=4; Student's *t*-test; *P*=0.0183). (**c** and **d**) HuH-6 cells were transfected with different combinations of TUG1 and miR-34a-5p mimic. qRT-PCRs were conducted to detect VEGFA expression. (+) corresponds to 50 ng TUG1 construct or 25 ng of miR-34a-5p mimic. (++) corresponds to 100 ng TUG1 construct or 50 ng of miR-34a-5p mimic. Data were shown as mean±S.E.M., and expressed as the relative change compared with the control group (*n*=4; Student's *t*-test). * indicated the significant difference between the marked groups. (**e**) HuH-6 cells were transfected with miR-34a mimic, miR-34a mimic+TUG1, or left untreated (Wt) for 24 h. Western blots were conducted to detect VEGFA expression. Tubulin was detected as the internal control. VEGFA expression was detected as the ratio of densitometric value compared with Tubulin expression. A representative immunoblot was shown along with the quantitative data (*n*=4; Student's *t*-test; *P*=0.0098). (**f**) HuH-6 cells were transfected with miR-34a mimic, scrambled miRNA mimic or left untreated (Wt) for 24 h. qRT-PCRs were conducted to detect TUG1 levels (*n*=4; Student's *t*-test; *P*=0.0101)

We employed PicTar database to predict the potential mRNA targets of miR-34a-5p.^16^ Among these putative targets, we focused on VEGFA, a key angiogenic factor involved in pathological angiogenesis (Figure 5a). The 3′-UTR of VEGFA was cloned into the luciferase coding region (RLuc-VEGF-WT) and transfected into HuH-6 cells with miR-34a-5p mimic or a negative control mimic. Luciferase assays revealed that VEGFA was a target of miR-34a-5p. The use of mutant derivatives (-Mut) in the miRNA recognition site further verified the specificity of inhibitory effect (Figure 5b).

If TUG1 effectively functions as a decoy, one would expect that the relative concentration of the decoy and miRNAs affects target gene expression. We gradually increased miR-34a-5p amount in the presence or absence of TUG1. TUG1 overexpression significantly increased VEGFA mRNA level, and was gradually reduced when miR-34a-5p level was increased (Figure 5c). We also gradually increased TUG1 amount in the presence or absence of miR-34a-5p. miR-34a-5p significantly decreased VEGFA mRNA level, whereas the decrease was partially restored when TUG1 level was increased (Figure 5d). Western blot analysis showed that miR-34a mimic transfection significantly downregulated VEGFA level, whereas TUG1 overexpression could partially rescue VEGFA downregulation (Figure 5e). We also found that miR-34a mimic transfection significantly downregulated endogenous TUG1 level (Figure 5f). Collectively, these results suggest that there is interplay among lncRNA-TUG1, miR-34a-5p and VEGFA.

### LncRNA-TUG1-miR-34a-5p-VEGFA network is critical for hepatoblastoma cell function

**Figure 6:**
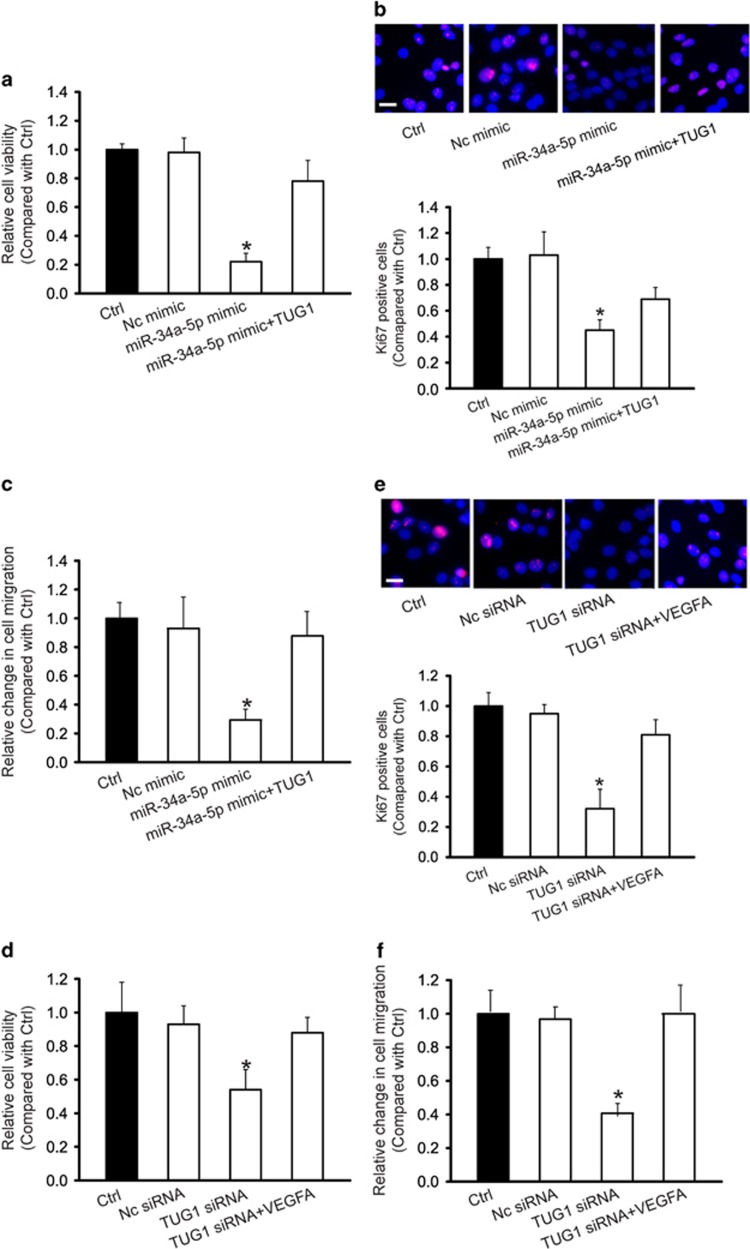
LncRNA-TUG1-miR-34a-5p-VEGFA network is critical for hepatoblastoma cell function. HuH-6 cells were treated as shown. Cell viability was detected using MTT assay (*n*=4; Student's *t*-test; *P*=0.0176 (**a**) and *P*=0.0366 (**d**)). A representative image for Ki67 staining along with quantification data was shown. Scale bar: 10 *μ*m *(n*=4; Student's *t*-test; *P*=0.0249 (**b**) and *P*=0.0216 (**e**)). Transwell assay was used to detect HuH-6 cell migration *(n*=4; Student's *t*-test; *P*=0.0207 (**c**) and *P*=0.0253 (**f**)). Data were shown as mean±S.E.M., and expressed as the relative change compared with the control group. *indicated significant difference compared with Ctrl group

We then determined whether TUG1-miR-34a-5p interaction is involved in regulating hepatoblastoma cell function. MTT assay, Ki67 staining, and transwell migration assay showed that miR-34a-5p mimic transfection significantly reduced the viability (Figure 6a), proliferation (Figure 6b), and migration (Figure 6c) of HuH-6 cells, whereas TUG1 overexpression partially countered these repressive effects. We also determined whether the TUG1-VEGFA interaction is involved in HuH-6 function. TUG1 knockdown significantly decreased the viability, proliferation, and migration of HuH-6 cells, whereas exogenous VEGFA treatment could partially reverse the effect of TUG1 knockdown (Figures 6d–f).

### Involvement of lncRNA-TUG1-miR-34a-5p-VEGFA network in angiogenesis for hepatoblastoma

**Figure 7:**
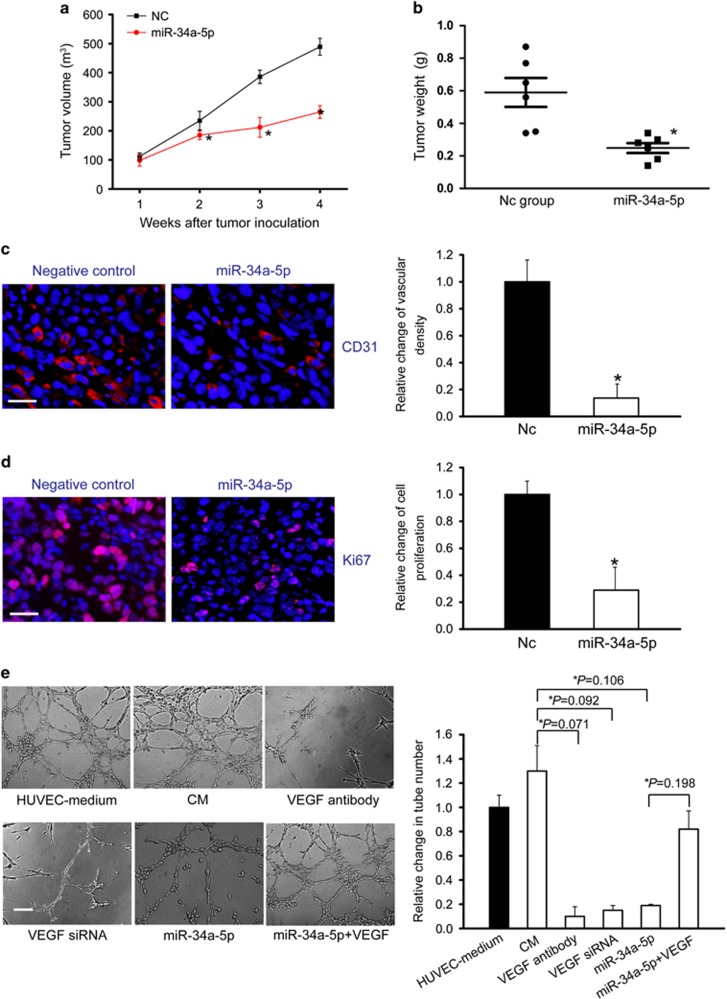
Involvement of lncRNA-TUG1-miR-34a-5p-VEGF network in angiogenesis for hepatoblastoma. (**a**) An *in vivo* hepatoblastoma model was established by injection of HuH-6 cells expressing the non-specific sequences or precursors of miR-34a-5p. Tumor growth was compared between miR-34a-5p-transfected group and Nc group. Tumor volume (*V*) was calculated using the formula, V=(*L* × W^2^) × 0.5 (*n*=6; Mann–Whitney *U*-test; *P*=0.0409 (2w), *P*=0.0207 (3w)*, P*=0.0199 (4w)). (**b**) The tumors were weighed immediately after isolation. The tumor weight was plotted between miR-34a-5p-transfected group and Nc group (*n*=6; Mann–Whitney *U*-test; *P*=0.0283). (**c** and **d**) CD31 and Ki67 immunofluoresence staining was conducted to detect microvascular density and cancer cell proliferation. A representative image and statistical result was shown ((*n*=6; Mann–Whitney *U*-test; *P*=0.0133 (**c**), *P*=0.0206 (**d**)). Scale bar: 100 *μ*m. (**e**) HUVECs were cultured in the following media: HUVEC-specific media as positive control; conditioned media (CM) of HuH-6 cells, CM pre-treated with VEGFA antibody; CM of HuH-6 cells in which the endogenous VEGF expression silenced by RNA interference; CM of HuH-6 cells ectopically expressing miR-34a-5p with or without recombinant VEGFA (*n*=4; Student's *t*-test). Tube formation was determined after 24 h of culture under a phase contrast microscope

To determine the role of miR-34a-5p in tumor angiogenesis *in vivo*, HuH-6 cells pre-transfected with non-specific sequences or precursors of miR-34a-5p were inoculated into the right dorsal flanks of nude mice. Compared with the control group, tumor volume was significantly reduced in miR-34a-5p-transfected group 2 weeks after the cancer inoculation (Figure 7a). At the end of experiment, tumor volume was reduced by 35% (*P*<0.01) in miR-34a-5p-transfected group (Figure 7a), which was consistent with tumor weight reduction in miR-34a-5p-transfected group (Figure 7b). Four weeks after inoculation, the xenograft tumors were removed to detect tumor angiogenesis. CD31 and Ki67 staining showed microvascular density and the proportion of proliferating (Ki67^+^) tumor cells significantly decreased in miR-34a-5p-transfected group (Figures 7c and d). Meanwhile, we found that compared with the control group, miR-34a-5p-transfected group had lower levels of TUG1 and plasma VEGFA (Supplementary Fig. S2).

miR-34a-5p downregulation-induced VEGF might be secreted from hepatoblastoma cells to activate vascular endothelial cells. To test the hypothesis that miR-34a-5p downregulation contributes to angiogenesis, we used HUVECs for tube-formation assays. Tube formation by activated HUVECs was achieved by supplementation with HUVEC-specific medium or the conditioned media (CM) of HuH-6 cells. Tube-formation activity of CM was abolished by pretreatment of CM with VEGF antibody, VEGF downregulation by RNA interference in donor cells, or miR-34a-5p upregulation in donor cells. The angiogenic activity of CM lost by miR-34a-5p upregulation was restored by exogenous VEGF administration (Figure 7e). The amount of VEGF in each CM was examined (Supplementary Fig. S3). Taken together, the angiogenic activity in CM of hepatoblastoma cells was primarily due to VEGFA production. TUG1-miR-34a-5p-VEGFA network is involved in tumor angiogenesis.

## Discussion

Hepatoblastoma is a common malignant hepatic tumor in children. Its metastasis is the primary obstacle to the development of efficient treatment.^8^ Angiogenesis is shown as an important component of metastatic pathway. The new vessels provide the main routes by which tumor cells exit the primary tumor site and enter the circulation.^17^ Here, we show that lncRNA-TUG1 knockdown inhibits tumor growth and tumor angiogenesis *in vivo*, and reduces hepatoblastoma cell viability, proliferation, invasion, and migration *in vitro*. LncRNA-TUG1 upregulation contributes to unusual hypervascularity of hepatoblastoma.

With the development of whole-genome and transcriptome-sequencing technologies, lncRNAs have received increasing attention.^18^ LncRNAs not only act as the intermediary between DNA and protein but also as important protagonists of many cell functions. Their deregulations are linked to several human disorders, especially in cancers.^19^ LncRNA dysregulation is associated with the recurrence, metastasis, and prognosis of cancer. lncRNAs could function as oncogenes, and promote matrix invasion of cancer cells and tumor growth.^20, 21^ The levels of TUG1 in hepatoblastoma tissues are significantly higher than those in the matched non-tumor tissues. Based on *in vivo* and *in vitro* evidence, we show that TUG1 upregulation contributes to unusual hypervascularity for hepatoblastoma progression. In previous studies, TUG1 dysregulation has been reported in many other cancers. TUG1 is upregulated in bladder cancer, gastric cancer and osteosarcoma,^22, 23, 24^ but is downregulated in human non-small cell lung cancer and in human glioma.^25, 26^ These results indicate that TUG1 may have a tissue-specific expression pattern. Thus, it is required to consider TUG1 tissue specificity when TUG1 is intervened for treating hepatoblastoma.

Increasing studies have revealed the intricate interplay among diverse RNA species, including messenger RNAs and non-coding RNAs. These RNA transcripts communicate with and co-regulate each other by competing for binding to the shared miRNAs.^13, 27^ LncRNA-TUG1, miR-34a-5p, and VEGFA mRNA constituted a competing endogenous RNAs (ceRNA) regulatory network. This regulatory network maintains a relative balance to avoid abnormal activation of hepatoblastoma cells. Once lncRNA-TUG1 is significantly upregulated during hepatoblastoma progression, TUG1 upregulation could alleviate miR-34a-5p repressive effect, resulting in a significant increase in the expression of VEGFA, a target gene of miR-34a-5p. In previous study, a similar regulatory mechanism of TUG1 has been reported. There was a reciprocal repression between TUG1 and miR-145 in an Argonaute2-dependent manner. TUG1-miR-145-ZEB2 constitutes a ceRNA regulatory network, and regulates epithelial to mesenchymal transition and radioresistance in human bladder cancer cells.^28^

miRNA-34a has been reported as tumor suppressor in the development of several cancers, such as neuroblastoma, cervical carcinoma, colon cancer, and prostate cancer.^17, 29, 30, 31^ It directly regulates the expression of proteins involved in cell cycle, differentiation, apoptosis, and antagonizes processes.^32, 33^ We reveal a role of miRNA-34a in hepatoblastoma progression. miRNA-34a can directly regulate both TUG1 and VEGFA expression. TUG1 overexpression may become a sink for miRNA-34a, thereby affecting the derepression of VEGFA. miRNA-34a is shown as a post-transcriptional regulator. TUG1 functions as a genome regulator at the transcriptional level. The regulatory loop integrates the transcriptional and post-transcriptional regulatory network involved in tumor growth and angiogenesis.

The importance of VEGF in pathological angiogenesis has been confirmed in numerous malignant, inflammatory, ischemic, infectious and immune disorders. VEGF family and its receptor system is shown as the critical regulator in angiogenesis signaling.^34, 35^ The production of VEGFA by the tumor affects the angiogenic switch, where new vasculature is formed in and around the tumor, allowing it to grow exponentially.^36^ LncRNA-TUG1 functions as a ceRNA to regulate VEGFA levels by sponging miRNA-34a. Thus, TUG1 regulation could alter VEGFA level, which in turn affects tumor angiogenesis.

In conclusion, we show that TUG1 level is significantly upregulated in liver samples from patients with hepatoblastoma and in the metastatic hepatoblastoma cell lines. TUG1 upregulation contributes to the hypervascularity of hepatoblastoma via VEGFA induction. TUG1 knockdown significantly suppresses tumor angiogenesis, thereby inhibiting tumor growth. TUG1/miR-34a-5p/VEGFA network may become a candidate target for hepatoblastoma therapy.

## Materials and Methods

### Human tissue specimens and liver cell line

Human hepatoblastoma and adjacent non-tumor liver tissues were collected from 10 patients undergoing resection of hepatoblastoma in Children hospital, Fudan University, China. Informed consent was obtained from every patient. This study was approved by the Institute Research Ethics Committee in this hospital.

Tumor cell lines, HepG2, HuH-6, and SMMC-7221, and nonmalignant QSG-7701 and L01 hepatocytes were maintained in Dulbecco's Modified Eagle's medium supplemented with 10% fetal bovine serum (FBS) in a humidified incubator with 5% CO_2_ at 37 °C.

### TUG1 knockdown by siRNA transfection

Three individual siRNAs (siRNA TUG1-1, siRNA TUG1-2, and siRNA TUG1-3) and the negative control siRNA was purchased from Shanghai GenePharma Co., Ltd (Shanghai, China). Target sequences for TUG1 siRNAs were as follows: siRNA1, 5′-GTACGTGTCTTGGAAGTCT-3′ siRNA2, 5′-GCCAAATAACTGAAGCTAT-3′, and siRNA3, 5′-GTCTGCATTGAGGATATAG-3′. The negative siRNA was a scrambled sequence with no homology within mouse or human genome. siRNA duplexes were transfected into cells twice (48 h) within a gap of 24 h at the final concentration of 100 nm using lipofectamine RNAi max reagent (Life Technologies).

### Quantitative RT-PCR (qRT-PCR)

Total RNAs were extracted using TRIzol reagent (Invitrogen, Carlsbad, CA, USA) and cDNAs were synthesized using a High Capacity cDNA Reverse Transcription Kit (Applied Biosystems, Foster City, CA, USA). qRT-PCRs were conducted using the Power SYBRs Green PCR Master Mix (Applied Biosystems) on the StepOne Plus Real-Time PCR System. The PCR profile was 94 °C for 10 min, and 42 cycles of 94 °C for 10 s and 58 °C for 15 s. GAPDH was used as internal control. The PCR products were verified by melting curve analysis as well as by 2.0% agarose gel electrophoresis. Data analysis was performed using the 2^−△△Ct^ method. The primer sequence was shown below: Forward sequence for mouse TUG1: 5′-GAGACACGACTCACCAAGCA-3′ Reverse sequence for mouse TUG1: 5′-GAAGGTCATTGGCAGGTCCA-3′ Forward sequence for human TUG1: 5′-ACGACTGAGCAAGCACTACC-3′ Reverse sequence for human TUG1: 5′-CTCAGCAATCAGGAGGCACA-3′.

### Cell viability detection

MTT assay was used to detect cell viability. In brief, cells were seeded at 1 × 10^4^ cells/well in a 96-well culture plate. After the required treatments, these cells were incubated with MTT (0.5 mg/ml) for 3 h at 37 °C. After medium removal, 100 mm DMSO solution was added to dissolve formazan crystal. The absorbance at 570 nm wavelength was detected using a microplate reader (Molecular Devices, Sunnyvale, CA, USA).

### TCM preparation

Tumor cells (1 × 10^5^) were transfected with required RNA oligonucleotides in a 12-well plate. Thirty-six hours after transfection, the medium was removed. Cells were washed with phosphate-buffered saline (PBS) three times, and then cultured for additional 12 h. The TCM was collected and centrifuged at 500 g to remove detached cells and then centrifuged at 12 000 × *g* to discard cell debris. TCM was then stored in aliquots at −80 °C until using for co-culture experiments.

### *In vitro* tube-formation assay

HUVECs were cultured in the medium containing 5% FBS and 1% penicillin/streptomycin or CM of HuH-6 cells. HUVECs (1 × 10^4^) were seeded into a 96-well culture plate pre-coated with Matrigel (BD Biosciences, San Jose, CA, USA) and then cultured in the indicated condition. After 24 h incubation, tube formation was observed using a phase contrast microscopy, and quantified by counting tube number. The levels of VEGFA in CM were detected using the ELISA kit.

### *In vivo* tumor angiogenesis assay

All animal experiments were performed in accordance with relevant institutional and national guidance and regulation. Stable TGU1 knockdown HuH-6 cells, scrambled shRNA-transfected HuH-6 cells, or HuH-6 cells stably transfected with miR-34a-5p precursor or non-specific miRNA sequence, were injected subcutaneously into the right flank of 4-week-old immunocompetent mice (C57BL/6 mice). Xenograft tumors were removed 4 weeks after inoculation and used for vascular density detection. Vascular density of tumor sections was determined using CD31 staining.

### TUG1 shRNA lentivirus construction

Three different TUG1 RNAi target sequences were as follows: 5′-GTGGTACAGCTCTCTAAGC-3′ 5′-GCCAAATAACTGAAGCTAT-3′ 5′-GTCTGCATTGAGGATATAG-3′. A scrambled RNAi with no homology within the mouse genome was used as the control (5′-CCGATCTGACATGACTGCG-3′). The lentivirus harboring these RNAi constructs was generated using the BLOCK-iT RNAi Expression System (Life Technologies, Grand Island, NY, USA) according to the manufacturer's instruction. Packaging and amplification of virus was conducted in HEK293T cells.

### Immunofluoresence assay

After the required treatment, HepG2 or HuH-6 cells were fixed with ice-cold methanol for 10 min at −20 °C. Cells were washed with PBS for three times. Non-specific binding sites were blocked with 5% BSA for 30 min. These cells were incubated with the primary antibody (Ki67, 1:200, Abcam, Cambridge, MA, USA) overnight at 4 °C, and then incubated with the secondary antibody conjugated with Alexa Fluor 594 (Invitrogen) for 3 h at room temperature, followed by incubation with 4′,6-Diamidino-2-phenylindole dihydrochloride (DAPI, Sigma-Aldrich, St. Louis, MO, USA) for 5 min. These cells were observed using a fluorescent microscope.

### ELISA

TCM was used for ELISA to detect VEGFA. TCM was diluted with a ratio of 1:1 with the sample dilution buffer, and the concentration of VEGFA in TCM was detected using VEGFA ELISA kit (R&D Systems, Minneapolis, MN, USA) according to the manufacturer's protocol. Optical density was detected using a microplate reader (Molecular Devices) at 450 nm. Each assay was carried out in triplicate, and repeated at least three times. Standard curves were created using purified VEGFA.

### Western blot

Equal amounts of protein samples (50 *μ*g) were separated by 12% SDS-PAGE and transferred to PVDF membrane. After blocking, the membranes were incubated with the primary antibodies at 4 °C overnight. After washing, the membranes were incubated with the secondary antibody (1:5000) for 1 h at room temperature. Protein signaling was detected using the enhanced chemiluminescence kit (Pierce, Rockford, IL, USA).

### Cell invasion and migration assay

Twelve-well culture plates with 8-*μ*m micropore inserts were used for cell invasion and migration assays. For cell invasion assay, the top side of the insert was coated with Matrigel (BD Biosciences). After the required treatment, hepatoblastoma cells were placed into the upper well, cultured for 24 h and allowed to invade into the Matrigel layer. For migration assay, hepatoblastoma cells were placed into the upper well, cultured for 24 h, and allowed to invade through the transwell plate. The cells on the inserts were fixed with 3% paraformaldehyde, stained with crystal violet, and counted using a light microscope. Invasion or migration was reported as the fold change in number of cells invading or migrating through the transwell plate.

### Statistical analysis

Data were expressed as means±S.E.M. from at least three independent experiments. Comparison between any two groups was by two-tailed unpaired *t-*test for normally distributed data or non-parametric Mann–Whitney *U*-test for non-normally distributed data. A probability value *P*<0.05 was considered statistically significant.

## Abbreviations

lncRNA: long non-coding RNA
ceRNA: competing endogenous RNA
CM: conditioned media
ELISA: enzyme-linked immunosorbent assay
shRNA: short hairpin RNA
siRNA: short interfering RNA
VEGF: vascular endothelial growth factor
HUVEC: human umbilical vein endothelial cell
RISC: RNA-induced silencing complex
miRNA: microRNA
GAPDH: glyceraldehyde-3-phosphate dehydrogenase
