# Supplementary material for: Targeting long non-coding RNA-TUG1 inhibits tumor growth and angiogenesis in hepatoblastoma
Source: Cell Death Dis. 2016 Jun 30;7(6):e2278–. doi: 10.1038/cddis.2016.143 (PMC5108331; doi:10.1038/cddis.2016.143)
Supplement: Supplementary Figures [file cddis2016143x1.pdf]

Supplementary figure legend

Figure S1 LncRNA-TUG1 knockdown results in decreased cell viability, proliferation, invasion, and migration of HepG2 cells

(A) HepG2 cells were transfected with scrambled (Scr) siRNA, TUG1 siRNA, or left untreated for 48 h. Cell viability was detected using MTT method (A). Ki67 immunofluorescence staining and quantitative analysis showed that TUG1 knockdown reduced HepG2 cell proliferation (B). Transwell assays showed that TUG1 knockdown reduced HepG2 cell migration and invasion (C and D). n=6; Student's *t*-test; *P*=0.0199 (A), *P*=0.0237 (B), *P*=0.0283 (C), *P*=0.0361 (D).

Figure S2 Detection of lncRNA-TUG1 and VEGFA level in hepatoblastoma model

An in vivo hepatoblastoma model was established by the injection of HuH-6 cells expressing the non-specific sequences or precursors of miR-34a-5p. Four weeks after inoculation, qRT-PCRs were conducted to detect TUG1 expression in xenograft tumors (A). ELISAs were conducted to detect VEGFA levels in plasma (B). n=4; Student's *t*-test.

Figure S3 VEGF amount in the conditioned media of HuH-6 cells used for in vitro tube formation assays

VEGF levels were detected using ELISAs (n = 4). The experimental groups include: CM of HuH-6 cells; CM before pretreatment with VEGF antibody; CM of HuH-6 cell with VEGF knockdown by RNA interference; CM of HuH-6 cells with ectopic expression of miR-34a-5p; CM of HuH-6 cells with ectopic expression of

miR-34a-5p supplemented with recombinant VEGF. n=6; Student's *t*-test; *P*=0.0126 (A), *P*=0.0298 (B).

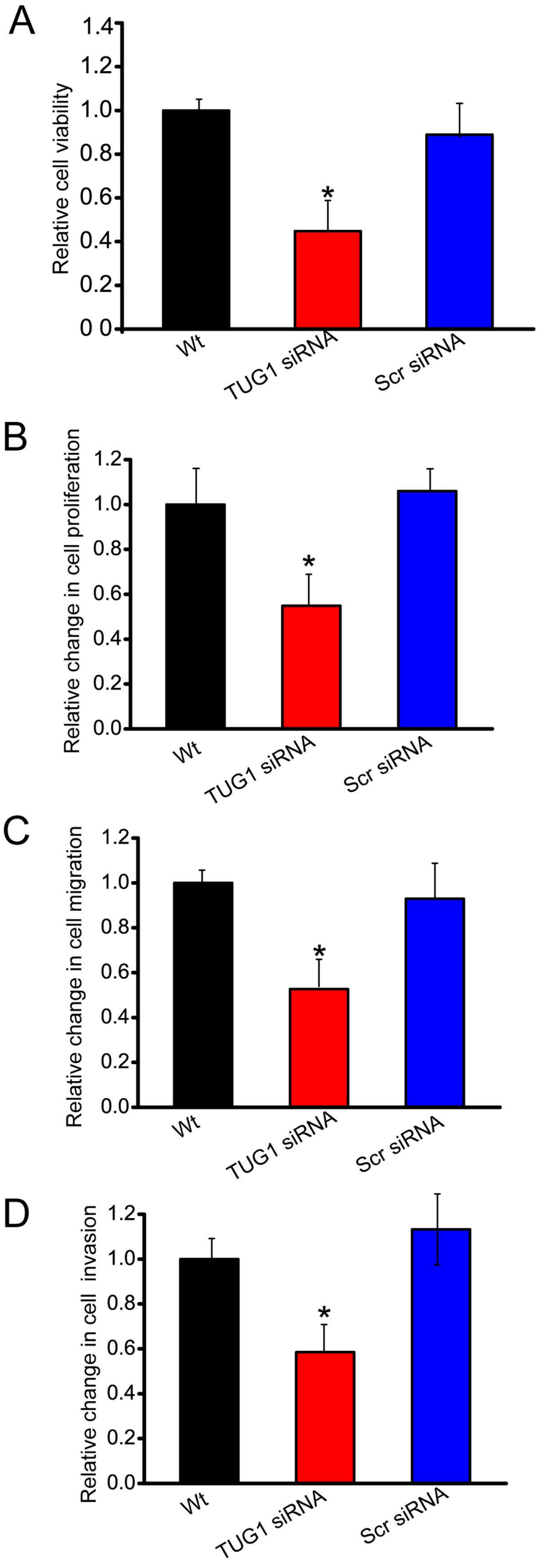
A
1.4
1.2
1.0
0.8
0.6
0.4
0.2
0.0
Relative cell viability
*
Wt
TUG1 siRNA
Scr siRNA
B
1.2
1.0
0.8
0.6
0.4
0.2
0.0
Relative change in cell proliferation
*
Wt
TUG1 siRNA
Scr siRNA
C
1.2
1.0
0.8
0.6
0.4
0.2
0.0
Relative change in cell migration
*
Wt
TUG1 siRNA
Scr siRNA
D
1.2
1.0
0.8
0.6
0.4
0.2
0.0
Relative change in cell invasion
*
Wt
TUG1 siRNA
Scr siRNA

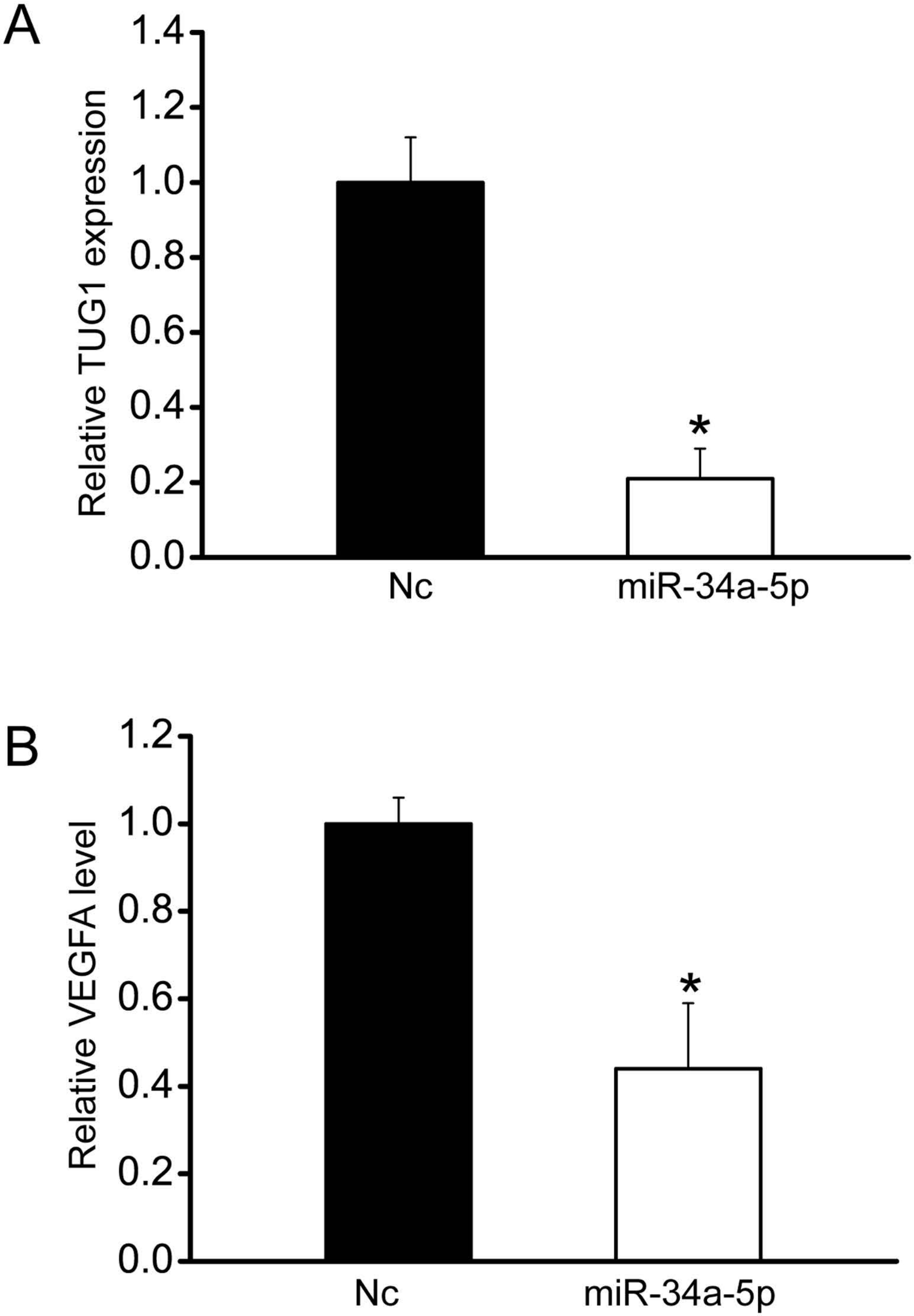
A
Relative TUG1 expression
1.4
1.2
1.0
0.8
0.6
0.4
0.2
0.0
*
Nc
miR-34a-5p
B
Relative VEGFA level
1.2
1.0
0.8
0.6
0.4
0.2
0.0
*
Nc
miR-34a-5p

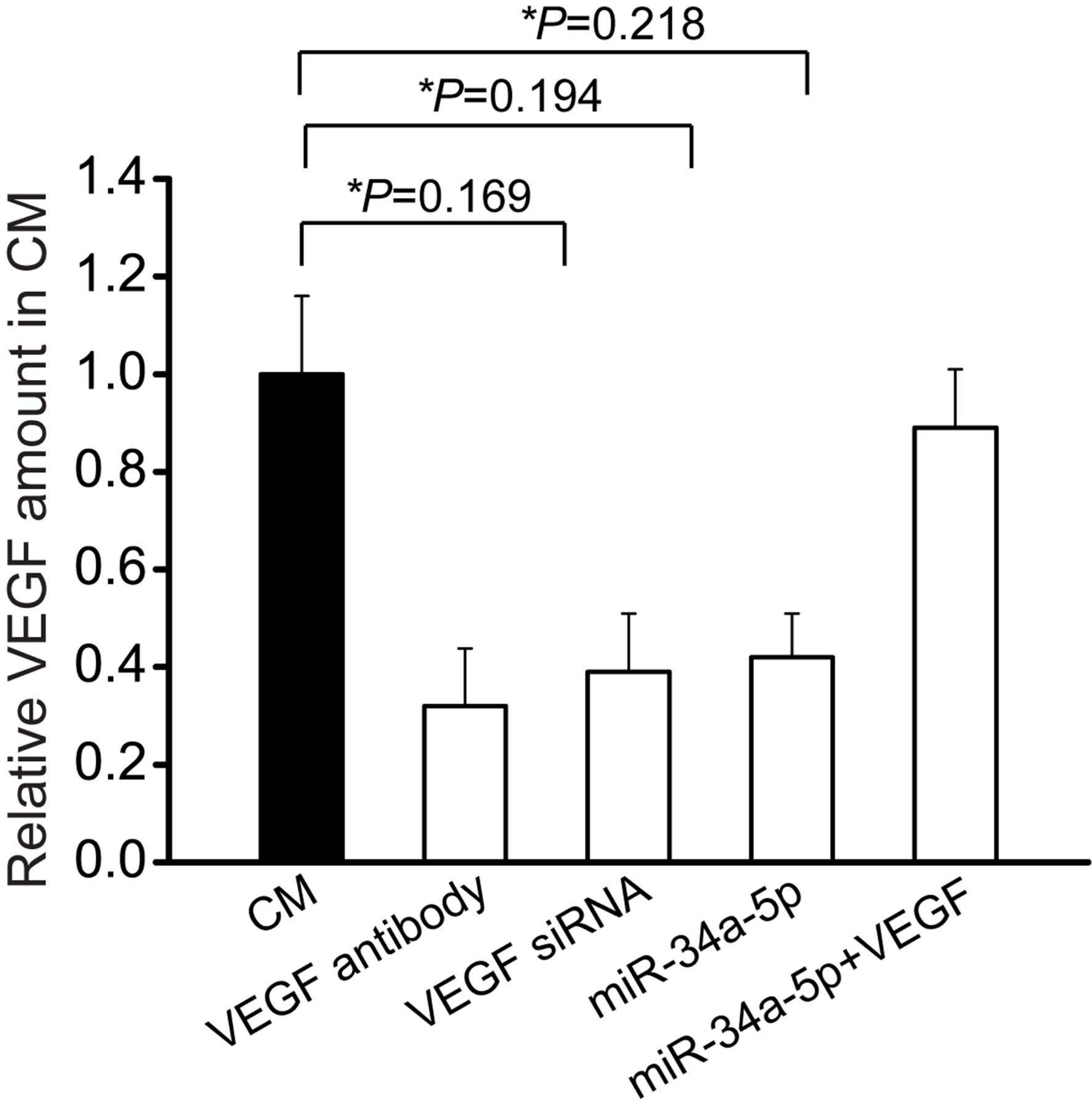
*P=0.218
*P=0.194
*P=0.169
Relative VEGF amount in CM
1.4
1.2
1.0
0.8
0.6
0.4
0.2
0.0
CM
VEGF antibody
VEGF siRNA
miR-34a-5p
miR-34a-5p+VEGF
